# Addressing Obesity in Special Populations

**DOI:** 10.1155/2014/171208

**Published:** 2014-09-18

**Authors:** Rachel A. Annunziato, Rachel M. Calogero, Robyn Sysko

**Affiliations:** ^1^Department of Psychology, Fordham University, Bronx, NY 10458, USA; ^2^Department of Psychology, University of Kent, Canterbury CT2 7NP, UK; ^3^Department of Psychiatry, New York State Psychiatric Institute, College of Physicians and Surgeons, Columbia University, New York, NY 10032, USA

Despite a substantial body of research, including the development of interventions to curb the alarming prevalence of obesity, little progress has been realized. With notable rates of overweight and obesity across the globe, a number of special populations have been identified among the larger population of obese individuals. As many of these special populations are understudied, the effectiveness of standard approaches for weight reduction is largely unknown, as is whether these strategies are congruent with the needs of diverse clients. Therefore, we are pleased to present six papers dedicated to this topic, four empirical pieces and two review articles.

The first review paper, by A. E. Bombak, offers a social sciences' perspective on the problem of obesity, with a focus on the role of stigma in obesity. The framework and recommendations provided by this contribution echo the empirical pieces in this compilation. Our first empirical study, by P. Ferrada et al., examines surgical outcomes for obese patients and offers an important finding, namely, that weight alone was not a risk factor for mortality in the large sample investigated. Certainly, this manuscript suggests important, positive implications for the medical management of obese individuals.

In addition, this special issue contains complementary works focused on the considerations and needs of diverse populations who are far less represented in the obesity literature. S. L. Katz-Wise et al. present an analysis using the comprehensive National Longitudinal Study of Adolescent Health (Add Health) dataset on weight change over time among young adults and differences according to gender, race/ethnicity, and sexual orientation. The authors found interactions between these variables that were associated with different weight gain trajectories, findings that are suggestive of a need to consider all of these characteristics when addressing unhealthy weight gain among youth. And, as A. E. Bombak eloquently describes, the issue of stigma is likely very salient here as well.

B. Robles et al. conducted a study on health risks and healthy eating among a large sample of low income, predominantly Black and Latino adults in Los Angeles County. Using sophisticated statistical analyses, they determined that being female and having high self-efficacy were the strongest predictors of healthy eating. These findings have clear implications for identifying specific targets for interventions to improve the eating habits of underserved populations. A similar population is targeted in the manuscript by C. Davis et al., who present the development of a spirituality-based weight loss intervention and their subsequent recruitment and retention efforts within a largely Black and Latino community. Results showed that this approach was highly acceptable to this hard-to-reach population. Indeed, taken together these papers certainly offer culturally congruent approaches to improve health behaviors among diverse populations.

Finally, we have included a review piece by T. L. Tylka et al. that aims to critically evaluate two methods of working within patient care and public health: the* weight-normative approach* (emphasis on weight and weight loss when defining health and well-being) and the* weight-inclusive approach* (emphasis on viewing health and well-being as multifaceted while directing efforts toward improving health access and reducing weight stigma). This paper reviews the literature on both sides, uniquely with an ethical lens, and takes on a central question: why obesity interventions have not worked and what should be done going forward.

In general, overall, this special issue illuminates the importance of a multifaceted view on weight and the need to consider diverse perspectives, which encompass stigma, when considering research and practice. We hope that together this group of papers generates empirically based suggestions for advancing the field in regard to addressing obesity among these who have been less represented.

*Rachel A. Annunziato* *Rachel A. Annunziato* *Rachel M. Calogero* *Rachel M. Calogero* *Robyn Sysko* *Robyn Sysko*

